# Study on the Mechanism of *Ganoderma lucidum* Polysaccharides for Ameliorating Dyslipidemia via Regulating Gut Microbiota and Fecal Metabolites

**DOI:** 10.3390/biom16010153

**Published:** 2026-01-14

**Authors:** Wenshuai Wang, Rui Sun, Jianjun Zhang, Le Jia, Yuanjun Dong

**Affiliations:** 1 School of Social Development, University of Health and Rehabilitation Sciences, Qingdao 266113, China; wangwenshuai@uhrs.edu.cn (W.W.); sunrui@uhrs.edu.cn (R.S.); 2 College of Life Science, Shandong Agricultural University, Taian 271018, China; zhangjj@sdau.edu.cn

**Keywords:** dyslipidemia, fecal metabolites, *Ganoderma lucidum* polysaccharides, gut microbiota, untargeted metabolomics

## Abstract

In today’s world, unhealthy living habits have contributed to the rise in metabolic disorders like hyperlipidemia. Recognized as a popular edible and medicinal mushroom in China and various eastern nations, *Ganoderma lucidum* is a promising high-value functional and medicinal food with multiple biological activities. Our earlier research has demonstrated that *G. lucidum* polysaccharides (GLP) showed distinct lipid-lowering abilities by enhancing the response to oxidative stress and inflammation, adjusting bile acid production and lipid regulation factors, and facilitating reverse cholesterol transport through Nrf2-Keap1, NF-κB, LXRα-ABCA1/ABCG1, CYP7A1-CYP27A1, and FXR-FGF15 pathways, hence we delved deeper into the effects of GLP on hyperlipidemia, focusing on its structural characterization, gut microbiota, and fecal metabolites. Our findings showed that GLP changed the composition and structure of gut microbiota, and 10 key biomarker strains screened by LEfSe analysis markedly increased the abundance of energy metabolism, and cell growth and death pathways which were found by PICRUSt2. In addition, GLP intervention significantly altered the fecal metabolites, which enriched in amino acid metabolism and lipid metabolism pathways. The results of structural characterization showed that GLP, with the molecular weight of 12.53 kDa, consisted of pyranose rings and was linked by α-type and β-type glycosidic bonds, and its overall morphology appeared as an irregular flaky structure with some flecks and holes in the surface. Collectively, our study highlighted that the protective effects of GLP were closely associated with the modification of gut microbiota and the regulation of metabolites profiles, thus ameliorating dyslipidemia.

## 1. Introduction

Over the past few decades, occurrences of metabolic syndrome have risen significantly worldwide, such as obesity, hyperlipidemia, type 2 diabetes mellitus, cardiovascular disease, and non-alcoholic fatty liver disease [1]. Hyperlipidemia is a metabolic disorder that involves abnormally high blood lipid levels, and its occurrence and development is often in connection with obesity and diabetes [2,3]. The improvement of lifestyle plus medicine intervention is considered as the great treatment for hyperlipidemia, but the adverse effects and contraindications of the current hypolipidemic agents becomes an important restraining factor of a hyperlipidemia cure [4].

Recently, accumulating clinical and experimental research has demonstrated the significant impact of gut microbiota on the onset and advancement of human illnesses. The distinct alterations of gut microbiota composition have been noted in the chronic metabolic disorder including obesity, CVD, T2DM, hyperlipidemia, and so on [5,6,7,8]. The imbalanced intestinal flora disturbs the endogenous profiles of intestinal metabolites that are converted from diet, host metabolites, and microbial compounds in association, ultimately resulting in diseases jointly [9]. Research findings have indicated that gut microbiota and its metabolites influence cholesterol and lipid metabolism, inflammation, and immunity [10,11]. On the other hand, metabonomics, a popular methodology of detecting the small molecular substances in biological systems, is conducive to excavating underlying pathogenesis mechanisms and the following development of novel drugs through the identification of important biomarkers [12,13]. Therefore, the comprehensive investigation gut microbiota and metabolomics might be a productive way to identify disease pathogenesis and therapeutic targets.

As a kind of traditional edible and medicinal mushroom preciously in China and other eastern countries, *Ganoderma lucidum* has been extensively utilized to promote health and extend lifespan dating back several hundreds of years and is a promising high-value functional and medicinal food [14]. With the development of modern pharmacology, the pharmacological functions of *G. lucidum* have been further recognized and extended, such as anti-inflammation, anti-tumors, antihyperlipidemic, antihyperglycemic, antidiabetic, and anti-obesity activities [15,16,17,18]. Meanwhile, many bioactive components have been isolated from *G. lucidum* such as polysaccharides, triterpenes, proteoglycans, steroids, alkaloids, and lactones [19,20]. *G. lucidum* polysaccharides (GLP), the key bioactive component found in *G. lucidum*, have been demonstrated to possess prominent effects on preventing and treating various diseases. Jin et al. found that GLP could regulate intestinal barrier functions by modulating inflammatory cytokines such as IFN-γ, IL-2, and IL-4, increasing the gut microbiota richness which reflected by the Chao 1 index and decreasing the *Firmicutes*-to-*Bacteroidetes* ratio [21]. Li et al. found that *G. lucidum* polysaccharide (Gl-PS) lowered fasting blood glucose and lipid levels and raised body weight and serum insulin levels, thus producing antihyperglycemic and hypolipidaemic activities in diabetic mice [22]. In addition, the development of submerged fermentation culture techniques provides an assurance of the production of *G. lucidum* and the subsequent bioactive polysaccharides [23]. It is universally acknowledged that the biological activities of polysaccharides are closely associated with their structural characteristics, and the differences in chemical properties of polysaccharides could lead to the differences in their pharmacological action [24,25]. Therefore, it is of great importance to exploring the structural characterizations of polysaccharides for clarifying its structure–activity relationships as soon as possible.

Our earlier research has demonstrated that GLP showed remarkable lipid-lowering abilities by relieving oxidative stress and inflammation state, regulating the metabolism of bile acids, promoting reverse cholesterol transport, and modulating the expressions of lipid regulatory factors, thus reducing lipid accumulation and maintaining cholesterol homeostasis [26]. In this work, we further probed into the effects of GLP on the high-fat diet (HFD)-induced dyslipidemia from the point of view of gut microbiota composition and fecal metabolite profiles. In addition, the structural characterization of GLP was investigated by means of the various methods, aiming to better elucidate the complicated relationship between structure and bioactivity.

## 2. Materials and Methods

### 2.1. Preparation of GLP

The GLP was obtained as described in our previous report [26]. In brief, mycelia polysaccharides from *Ganoderma lucidum* were preliminarily obtained through the water extraction and alcohol precipitation technique. Subsequently, after being treated by deproteinization, depigmentation, dialysis, and purified by DEAE-52 cellulose column, the GLP was prepared successfully.

### 2.2. Structural Characterization of GLP

Gold powders were sputtered onto the samples, which were then examined using a field emission scanning electron microscope (SEM, SU8020, Hitachi Limited, Tokyo, Japan) to determine the shape and surface morphology of GLP.

The GLP was dissolved in DMSO solution at the concentration of 1 mg/mL and filtered through a filter of 0.45 μm. Subsequently, the molecular weight was measured using gel permeation chromatography (GPC) equipped with three tandem columns (300 × 8 mm, Ohpak SB-805 HQ, Ohpak SB-804 HQ and Ohpak SB-803 HQ; Showa Denko K.K., Tokyo, Japan) at 60 °C with the flow rate of 0.3 mL/min. The absolute molecular weights were calculated by Optilab T-rEX differential refractive index (RI) detector (Wyatt technology, Goleta, CA, USA) and DAWN HELEOS II multi-angle light scattering (MALS) detector (Wyatt technology, Goleta, CA, USA).

Fourier transform infrared spectrometry (FT-IR) is an important instrument which is extensively used for identifying the structure and composition of compounds. The powders of GLP were mixed with 200 mg KBr powders and pressed into 1 mm pellets and then measured using a spectrometer (Nicolet iZ-10, Thermo Nicolet, Madison, WI, USA) in the range of 4000–400 cm^−1^ with the resolution ratio of 4.00 cm^−1^.

The glycosidic linkages of GLP were elucidated via methylation analysis, with the detailed experimental procedure described as follows. GLP (2–3 mg) was dissolved in 500 μL dimethyl sulfoxide, followed by the addition of 1 mg sodium hydroxide, then the mixture was incubated for 30 min at room temperature. Subsequently, 50 μL iodomethane was added to initiate the methylation reaction, which proceeded for 1 h. Following extraction with dichloromethane, centrifugation, and evaporation of the organic phase under a stream of N_2_, the methylated GLP was collected. The methylated GLP was then hydrolyzed with 100 μL of 2 mol/L trifluoroacetic acid (TFA) at 121 °C for 90 min. Residual TFA was removed by evaporation under reduced pressure at 30 °C. The hydrolyzed products were reduced with sodium borodeuteride in 50 μL of 2 mol/L aqueous ammonia solution at room temperature for 2.5 h, and the reduction reaction was terminated by the addition of glacial acetic acid. Subsequently, 250 μL acetic anhydride was added, and the acetylation reaction was carried out at 100 °C for 2.5 h. Finally, the resulting partially methylated alditol acetates (PMAAs) were extracted with methylene chloride and analyzed using a gas chromatography-mass spectrometer (GC-MS, 7890A-5977B, Agilent Technologies Inc. Santa Clara, CA, USA).

The polysaccharide samples were dissolved in D_2_O, and subsequently the 1D and 2D nuclear magnetic resonance (NMR) spectra of GLP were recorded at 25 °C by a NMR spectrometer (Bruker, Rheinstetten, Germany) at 600 MHz, including ^1^H, ^13^C, mononuclear chemical shift correlation spectroscopy (COSY), nuclear Overhauser effect spectroscopy (NOESY), heteronuclear single-quantum coherence (HSQC), and heteronuclear multiple bond correlation (HMBC) spectra.

### 2.3. Animals Experiment Design

**Figure 1:**
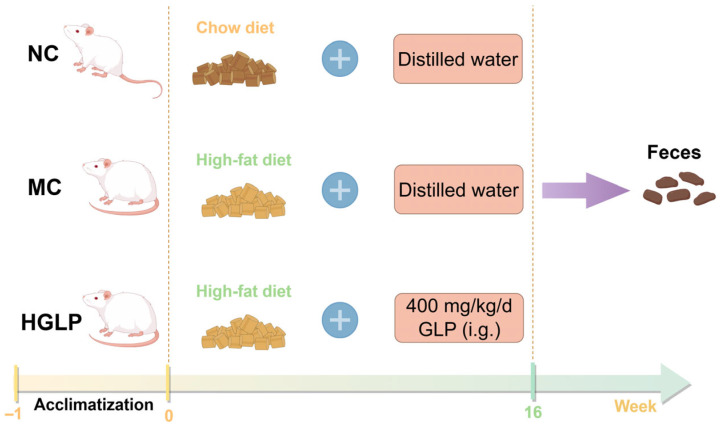
The schedule of the animal experiments.

In accordance with our previous report [26], after adaptive feeding for 1 week, the male Kunming strain mice, weighing between 18 and 22 g, were randomly assigned to three different treatment groups (10 mice in each group) and fed for 16 weeks: (1) the normal control (NC) group treated with a normal chow diet, (2) the model control (MC) group fed with HFD, and (3) the HGLP group fed with HFD plus 400 mg/kg/d GLP by gavage. At the end of the experiment, the feces samples of mice were collected and stored in a −80 °C refrigerator for later use. The animal experiment schedule is shown in Figure 1.

### 2.4. 16S rRNA Sequencing and Analysis

The gut microbiota was determined by 16S rRNA sequencing [27,28,29]. The microbial genomic DNA of fecal samples was extracted using the MagPure Soil DNA LQ Kit (Magen), and then the V3-V4 hypervariable region of the bacterial 16S rRNA gene was amplified using primers B341F (5′-CCTACGGGNGGCWGCAG-3′) and B785R (5′-GACTACHVGGGTATCTAATCC-3′). The PCR products were confirmed by 2% agarose gel electrophoresis and recovered using QIA quick Gel Extraction Kit (Qiagen). After quantified by the KAPA Library Quantification Kit (KAPA Biosystems, Wilmington, MA, USA), the sequencing libraries were paired-end sequenced on Illumina Novaseq 6000 sequencing platform. Afterwards, all the sequences were classified by the operational taxonomic units (OTU) with a similarity cutoff of 97%. Finally, gut microbial abundance and diversity were assessed through alpha (α) diversity and beta (β) diversity analysis. Linear discriminant analysis (LDA) effect size (LEfSe) was used to identify the significantly different bacterial taxa among groups with LDA score threshold > 2 and *p* < 0.05. Functional prediction analysis of microbiota was conducted by PICRUSt2. The 16S rRNA gene amplicon sequencing and analysis were performed through Sanshu Biotech Co., Ltd. (Nanjing, China).

### 2.5. Untargeted Metabolomics Analysis Based on LC-MS

Feces sample was mixed with methanol/acetonitrile solution (1:1, *v*/*v*), vortexed for 60 s and conducted with sonication at 4 °C for 30 min. After centrifugation (10 min, 12,000 rpm, 4 °C) treatment, the supernatants were dissolved in 200 μL of 50% acetonitrile (*v*/*v*) and centrifuged again at 14,000× *g*, 4 °C for 15 min, then the supernatants were prepared completely for detection. In addition, quality control (QC) samples were prepared by mixing aliquots of each sample and analyzed together with the other samples to validate system stability and repeatability.

The metabolites were determined using ultra-high performance liquid chromatography (UHPLC, Vanquish, Thermo, USA) coupled with a high-resolution mass spectrometer (MS, Q Exactive HFX, Thermo, USA) [29,30]. The raw MS data were processed using Progenesis QI 2.0 (Waters Corporation, Milford, MA, USA) software. Data were ulteriorly subjected to multivariate data analysis, including principal component analysis (PCA), partial least squares discriminant analysis (PLS-DA), and orthogonal PLS-DA (OPLS-DA). Metabolites with the variable importance for the projection (VIP) > 1 in the OPLS-DA model and *p*-value < 0.05 in Student’s *t*-test were considered as statistically significant, which were subjected to KEGG pathway analysis subsequently. The untargeted metabolomics analysis was performed through Sanshu Biotech Co., Ltd. (Nanjing, China).

### 2.6. Statistical Analysis

The data were expressed as mean ± standard deviation (SD), with *p* < 0.05 considered a statistically significant difference. Statistical analysis was conducted using one-way ANOVA and followed by the Duncan Test (SPSS 19.0 statistics software). Different letters of any two groups indicated significant differences (*p* < 0.05).

## 3. Results

### 3.1. Structural Characterization

**Figure 2:**
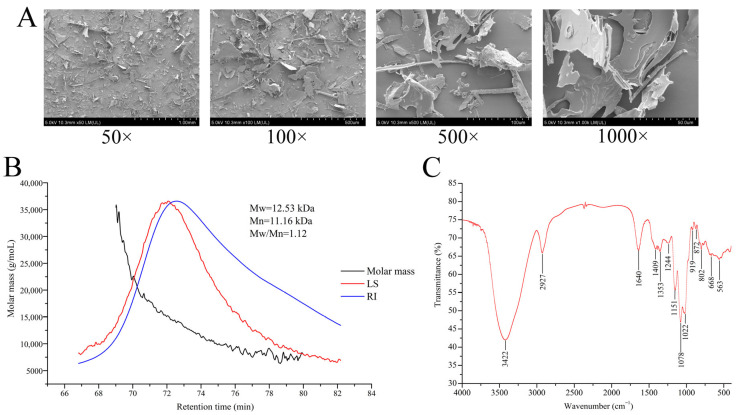
Structural analysis of GLP. (**A**) SEM images. (**B**) Molecular weight of GLP measured by GPC. (**C**) FT-IR spectrum. LS was the signal of multi-angle light laser scattering. RI was the signal of refractive index detector.

According to Figure 2A of SEM images, under the low magnification (50× and 100×), the overall morphology of GLP was an irregular lamellar structure of different sizes with no curls, and there were some small fragments scattered on its surface. At the higher magnifications (500× and 1000×), the rough surface of GLP with some flecks and holes was revealed.

The GPC-RI-MALS analysis confirmed that the weight-average molecular weight (Mw) of GLP was 12.53 kDa, and the number-average molecular weight (Mn) was 11.16 kDa. In addition, the polydispersity index (Mw/Mn) was 1.12, manifesting a comparatively uniform molecular mass distribution in GLP (Figure 2B).

In the FT-IR spectrum, the typical characteristic absorption bands of polysaccharide at 4000–400 cm^−1^ were revealed (Figure 2C). The strong bond at 3422 cm^−1^ was assigned to the stretching vibration of O-H, and the absorption peak at 2927 cm^−1^ resulted from the stretching vibration of the C-H bond [31,32]. A peak was found at 1640 cm^−1^, which corresponded to the C=O stretching vibration. In addition, three peaks for C-O-C and C-O-H stretching vibrations in the 1200–1000 cm^−1^ range (1151, 1078, and 1022 cm^−1^) indicated the existence of pyranose rings in GLP [33]. The absorption peaks at 872 cm^−1^ and 919 cm^−1^ suggested the presence of α- and β-type glycosidic linkages in GLP [34].

Methylation analysis showed that GLP contained 11 types of glycosidic fragment forms, and their molar ratios are summarized in Table 1. Consistent with the FT-IR analyses, the 11 kinds of sugar residues of GLP were all pyranose rings. In the partly methylated mixture of GLP, the relative molar ratio of Gal, including 6-Gal(p), 2,6-Gal(p), 2-Gal(p), and t-Gal(p), took up 69.566% with the highest proportion, which was in line with the findings of the monosaccharide composition in our previous report [26]. The total relative molar ratio of 6-Glc(p), 4-Glc(p), 3-Glc(p), and 3,6-Glc(p) was 18.806%, which was basically in accordance with the 17.24% relative molar ratio of Glc in monosaccharide composition analysis. Nevertheless, the glycosidic residues generated from the monosaccharides were partially inconsistent with the monosaccharide composition analysis. In detail, the methylation products of Xyl, Rib, and GlcUA were not detected probably because their contents were too low. Meanwhile, Fuc has poor methylation efficiency because of its structure; therefore, its detectable methylation bond form is significantly lower than monosaccharide composition when it is higher than 10%.

**Figure 3:**
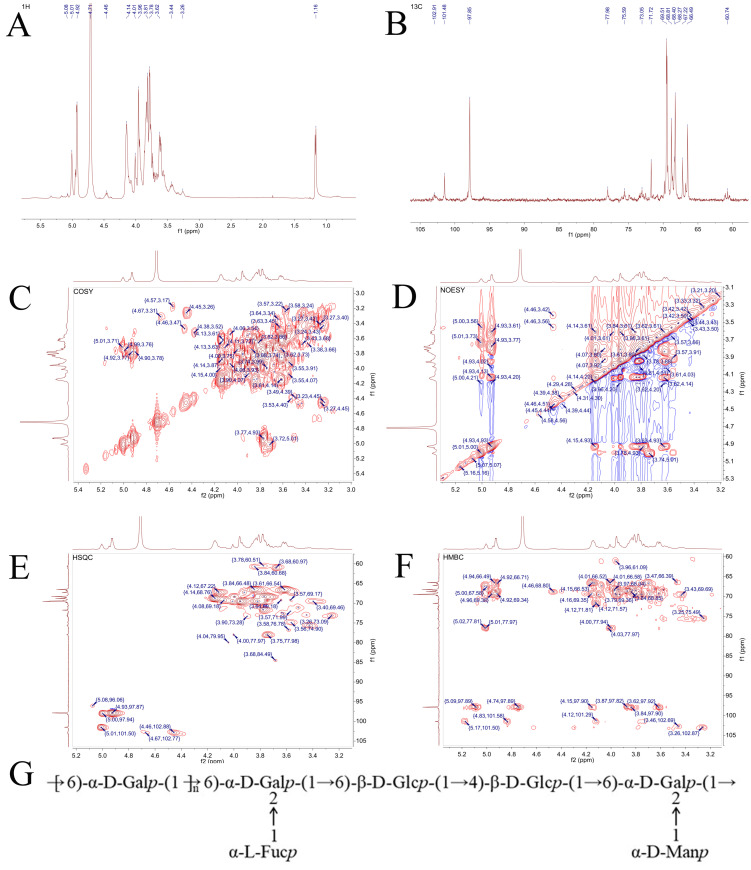
The NMR spectra of GLP including (**A**) ^1^H spectrum, (**B**) ^13^C spectrum, (**C**) COSY spectrum, (**D**) NOESY spectrum, (**E**) HSQC spectrum, and (**F**) HMBC spectrum. (**G**) Predicted structure of repeat units of GLP.

The 1D and 2D NMR spectra of GLP were illustrated in Figure 3A–F. In the ^1^H and ^13^C spectra, the anomeric proton and carbon signals of polysaccharides appeared in the regions of 4.3–5.8 ppm and 95–110 ppm, respectively. According to the 1D and 2D NMR spectra combined with the methylation analysis and published literature comprehensively, the chemical shifts in glycosyl residues in GLP were attributed in Table 2. Ulteriorly, the existing structure and glycosidic residues linkages of GLP were inferred. As displayed in Figure 3G, →6)-α-D-Gal*p*-(1→, little →6)-β-D-Glc*p*-(1→ and →4)-β-D-Glc*p*-(1→ connected and composed the main chain of GLP, while its branched chain was mainly composed of α-L-Fuc*p*-(1→ and α-D-Man*p*-(1→ attached to the O-2 site of →2,6)-α-D-Gal*p*-(1→.

### 3.2. The Effects of HGLP on HFD-Induced Dysregulation of Gut Microbiota

**Figure 4:**
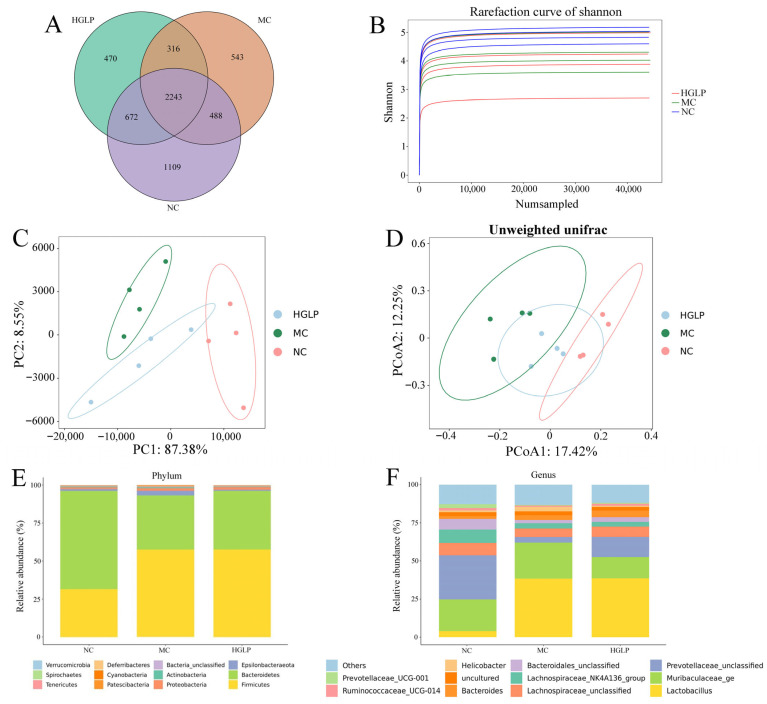
Effects of HGLP on the gut microbiota alterations in HFD-treated mice (n = 4 for each group). (**A**) Venn diagram showing the OTUs distribution of NC, MC, and HGLP groups. (**B**) Shannon rarefaction curve. (**C**) PCA analysis based on Euclidean distances. (**D**) PCoA analysis based on Unweighted unifrac distance matrix. (**E**) Relative abundance barplot of the gut microbiota at phylum level. (**F**) Relative abundance barplot of the gut microbiota at genus level.

In the study, sequencing of 16S rRNA was conducted to evaluate the effect of HGLP treatment on gut microbiota composition. The raw data was processed to obtain clean data, then OTUs were grouped with a similarity cutoff of 97%. Overall, a total of 5841 OTUs were obtained, with 2243 common OTUs in the three groups (Figure 4A). There were 4512, 3590, and 3701 OTUs in NC, MC, and HGLP group, respectively, which indicated that the abundances of flora were highest in the NC group and lowest in the MC group between the three groups. The community richness and diversity can be estimated by alpha diversity analysis, and the results indicated that no statistically significant differences were found in the alpha diversity indices across the three groups (Figure S1). The rarefaction curve of each sample was smooth, implying that the sequencing scope was capable of representing the samples’ flora characteristics (Figure 4B). In the beta diversity analysis, principal component analysis (PCA) based on Euclidean distances and principal coordinate analysis (PCoA) based on Unweighted unifrac distance were performed to assess the variation in bacterial community composition among different groups. As illustrated in Figure 4C,D, an apparent separation among the NC group and MC group was observed, demonstrating that HFD significantly changed the constructures of the gut bacterial communities. At the same time, the samples of the HGLP group were distributed between the samples of the NC and MC groups in the plots, which also indicated that HGLP could attenuate HFD-induced dysregulation of the intestinal flora in mice.

Subsequently, microbial composition at different taxonomic levels was investigated and illustrated in Figure 4E,F. At the level of phylum, the dominant microbes were Firmicutes and Bacteroidetes among the three groups, which accounted for the largest proportion of phyla. The relative abundance of Firmicutes was much higher, whereas the relative abundance of Bacteroidetes was much lower in the MC group than that in the NC group. The intervention of HGLP reduced the high ratio of Firmicutes to Bacteroidetes induced by HFD by increasing the abundance of Bacteroidetes, although it had no apparent effect on Firmicutes. In addition, a markedly increased abundance of Epsilonbacteraeota in the MC group was significantly decreased in the HGLP group (Figure 4E). At the genus level, a total of 167 genera were identified in the three groups, and bacteria with abundance greater than 1% were displayed in Figure 4F. The relative abundance of *Lactobacillus*, *Muribaculaceae_ge*, *Bacteroides*, and *Helicobacter* were increased after HFD feeding, while HGLP effectively reversed the changes in *Muribaculaceae_ge* and *Helicobacter*. When compared with the NC group, the MC group had significantly lower abundance in *Prevotellaceae_unclassified*, *Lachnospiraceae_unclassified*, *Lachnospiraceae_NK4A136_group*, *Bacteroidales_unclassified*, *Ruminococcaceae_UCG-014*, and *Prevotellaceae_UCG-001*, whereas these bacteria strains were increased in HGLP group except *Lachnospiraceae_NK4A136_group* (Figure 4F). These results revealed that HGLP treatment has the potential to rectify the imbalanced gut microbiota to some extent induced by HFD at different taxonomic levels.

### 3.3. Screening of Differential Flora and Analysis of Microbial Functional Pathways

**Figure 5:**
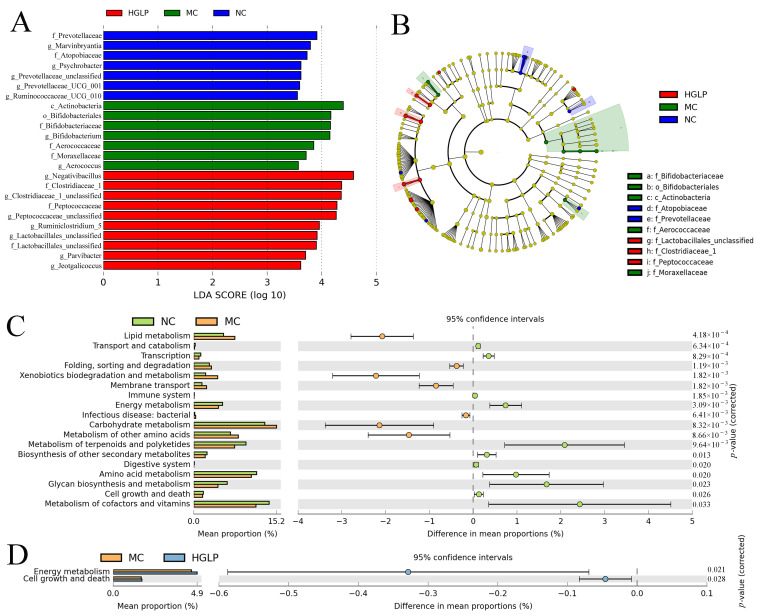
Core bacterial phenotype analysis based on LEfSe including (**A**) LDA scores histogram of taxa enriched at different taxonomy levels and (**B**) Taxonomic cladogram showing the significantly enriched taxa. Predictive functional profiling of microbial communities in level 2 of KEGG pathways by PICRUSt2 including KEGG pathway difference analysis between (**C**) NC group and MC group, as well as (**D**) MC group and HGLP group.

To identify specific differential gut bacteria as key biomarkers between groups, LEfSe was utilized, with criteria set at an LDA score above 2 and a *p*-value below 0.05. The results showed that the MC group was enriched with Actinobacteria, Bifidobacteriales, Aerococcaceae, Bifidobacteriaceae, Moraxellaceae, *Aerococcus*, and *Bifidobacterium*. The significantly enriched stains in the HGLP group were Peptococcaceae, Clostridiaceae_1, Lactobacillales_unclassified, *Peptococcaceae_unclassified*, *Clostridiaceae_1_unclassified*, *Parvibacter*, *Negativibacillus*, *Jeotgalicoccus*, *Lactobacillales_unclassified*, and *Ruminiclostridium_5*, suggesting that the above flora maybe play a crucial part in the intestinal flora homeostasis of HGLP against dyslipidemia (Figure 5A,B).

We further employed PICRUSt2 to predict the functional profiling of intestinal flora in the level 2 of the KEGG pathway. As illustrated in Figure S2 and Figure 5C,D, HFD feeding profoundly increased the microbiota related to cardiovascular disease, lipid metabolism, and neurodegenerative disease, but functional abundance of flora in energy metabolism, amino acid metabolism, transport and catabolism, translation, transcription, biosynthesis of other secondary metabolites, and other pathways were reduced. Compared to the MC group, mice in the HGLP group exhibited a microbiome with a notably higher number of functional genes for energy metabolism and cell growth and death.

### 3.4. GLP Modulated the Overall Fecal Metabolite Profile in Hyperlipidemic Mice

**Figure 6:**
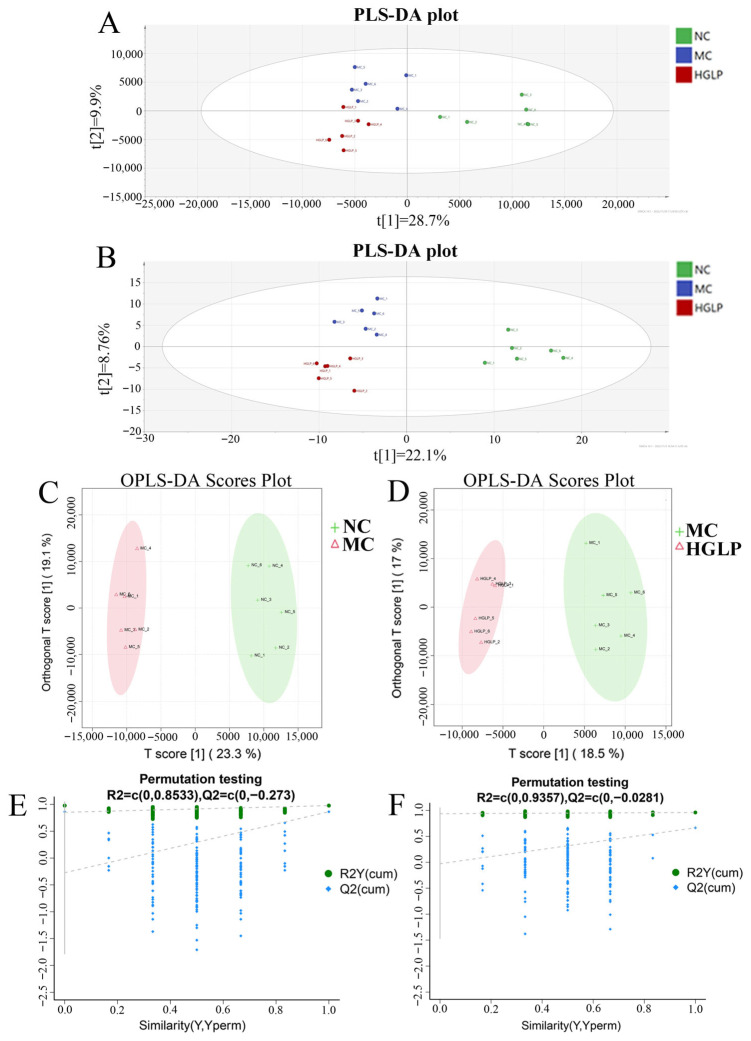
HGLP altered the overall metabolite profile induced by HFD (n = 6 for each group). PLS-DA score plot in (**A**) positive ion mode and (**B**) negative ion mode. (**C**) OPLS-DA score plot between MC and NC groups. (**D**) OPLS-DA score plot between HGLP and MC groups. (**E**) 200-response permutation testing plot of the OPLS-DA model in MC and NC groups. (**F**) 200-response permutation testing plot of the OPLS-DA model in HGLP and MC groups.

In the positive (ESI+) and negative (ESI-) ion modes, a total of 1619 metabolites were identified and quantified. To intuitively unveil the overall metabolic changes between samples from distinct groups, we adopted the multivariate statistical analysis to characterize metabolic disorders during the dyslipidemia process, including PLS-DA and OPLS-DA approaches. In the PLS-DA score plot, obvious separations between the samples in the NC, MC, and HGLP groups, indicating the different metabolite expression pattern of samples (Figure 6A,B). In comparison to PLS-DA, OPLS-DA reduces the influence of irrelevant interference factors, thus better distinguishing the inter-group differences and improving the model’s efficiency and analytical capacity. As shown in Figure 6C,D, significant intra-group clustering and clear inter-group separation were observed among the MC vs. NC and HGLP vs. MC groups, which manifested that HFD led to the apparent alterations in fecal metabolites, while HGLP had evident effects on modulating the metabolic disorders arisen from HFD. The reliability of OPLS-DA model could be verified by 7-fold cross-validation method, and the model evaluation parameters including R2Y (cum) and Q2 (cum) were 0.96 and 0.665 in the HGLP vs. MC groups, and 0.978 and 0.863 in the MC vs. NC groups, respectively. The results showed that the OPLS-DA models were stable and reliable. Moreover, the blue regression line of the Q2-points intersected the vertical axis (on the left) below zero in the 200-response permutation testing, which indicated that the OPLS-DA models had good fit and predictive capability and could be used for screening differential markers (Figure 6E,F).

By setting a VIP threshold of more than 1 in the OPLS-DA and a *p*-value of less than 0.05 (Student’s *t*-test), 131 differential metabolites were identified between the MC group and NC group, primarily falling under carboxylic acids and derivatives, fatty acyls, and benzene and substituted derivatives. Meanwhile, 44 metabolites were significantly changed in HGLP group compared with the MC group, mainly including carboxylic acids and derivatives, steroids and steroid derivatives, and fatty acyls (Tables S1 and S2, Figure S3). Above results also verified that the disturbance of HFD and the regulation of GLP on the lipid metabolism in fecal metabolite profile.

In detail, compared with the NC group, the MC group exhibited 73 significantly upregulated and 58 significantly downregulated differential metabolites. When compared with the MC group, the HGLP group showed 22 significantly increased and 22 significantly decreased differential metabolites. Additionally, hierarchical clustering heatmap analysis was conducted to intuitively visualize the relative expression levels of these differential metabolites in each sample across the NC, MC, and HGLP groups (Figure S4). These results further demonstrate that HGLP can alleviate fecal metabolite disorders triggered by HFD-induced dyslipidemia to a certain extent.

### 3.5. The Effect of GLP on Metabolic Pathways Regulation in Fecal Metabolites

**Figure 7:**
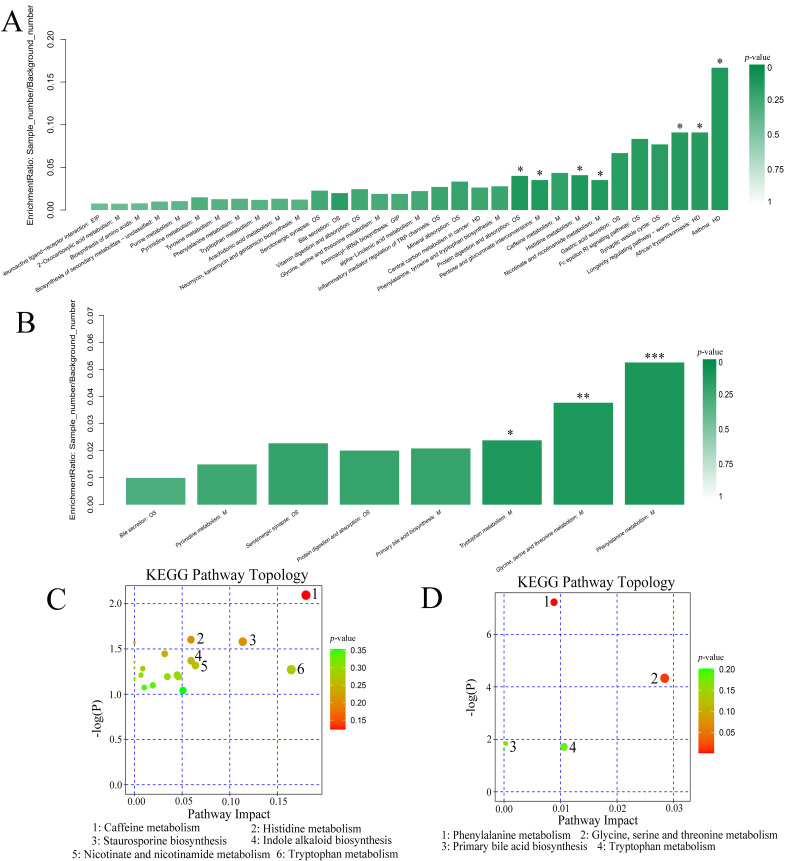
Metabolic pathway analysis among the three groups. KEGG pathway enrichment analysis between the (**A**) MC vs. NC groups and (**B**) HGLP vs. MC groups. The important metabolism pathway bubble plots of KEGG topological analysis between the (**C**) MC vs. NC groups and (**D**) HGLP vs. MC groups. * indicated that *p*-value < 0.05, ** indicated that *p*-value < 0.01, and *** indicated that *p*-value < 0.001.

To clarify the changes in metabolic pathways, KEGG pathway enrichment and topological analyses were conducted. The differential metabolites among the NC group and MC group enriched in 32 pathways. There were eight enriched pathways containing differential metabolites between HGLP and MC groups including glycine, serine and threonine metabolism, phenylalanine metabolism, tryptophan metabolism, primary bile acid biosynthesis, bile secretion, pyrimidine metabolism, protein digestion and absorption, and serotonergic synapse (Figure 7A,B). In the MetPA analysis, the six important potential metabolic pathways in the MC group were identified as caffeine metabolism, tryptophan metabolism, staurosporine biosynthesis, nicotinate and nicotinamide metabolism, histidine metabolism, and indole alkaloid biosynthesis when compared with NC group, while HGLP intervention significantly regulated four metabolic pathways including phenylalanine metabolism, glycine, serine and threonine metabolism, primary bile acid biosynthesis, and tryptophan metabolism (Figure 7C,D). The aforementioned pathways could serve as potential mechanisms for pathogenesis and therapeutic targets, with the differential metabolites in these crucial metabolic pathways likely being key biomarkers.

## 4. Discussion

Found extensively in organisms, especially within plant or fungal cells, polysaccharides are biological macromolecules consisting of monosaccharides joined by glycoside bonds [33]. In recent years, fungal polysaccharides have been confirmed to possess various biological activities and have been made into tablets and injections for the clinical use as adjuvant therapy for cancer chemotherapy [32,35]. The biological activities of polysaccharides are closely related to their structural characterization including molecular weight, conformation, functional groups, and branching [36]. Different methods of separation and purification have certain effects on the efficacy and characteristics of polysaccharides [37]. Accordingly, the structure characterization of GLP was analyzed. We found that GLP was composed of fucose, galactose, glucose, xylose, mannose, ribose, and glucuronic acid, and galactose was the main component in the backbone of GLP [26]. In addition, mannose and galactose are recognized as the monosaccharides associated with biological activity [38]. There was a quite difference between our findings and the reports of Fu et al. and Xu et al., which may be related to extraction site and extraction conditions [39,40]. As a useful tool for visualizing the morphology of polysaccharides, SEM analysis exhibited an irregular flaky structure with some flecks and holes in the unsmooth surface of GLP, which was similar to the small pieces with irregularity, fleckiness, and many holes on the surface of polysaccharides reported by Huang et al. [41]. Currently, plentiful studies on the structure of *G. lucidum* polysaccharides showed that its molecular weight is mostly distributed in the range of 10^3^–10^6^ Da. In the present study, the molecular weight of GLP was determined via GPC-RI-MALS to be 12.53 kDa, exhibiting no significant discrepancy from the results reported by Bao et al. [42] and Ye et al. [43]. It is widely recognized that the types of glycosidic bonds are intimately associated with the biological activities of polysaccharides. Our current investigation demonstrated that GLP was composed of pyranose rings connected via both α- and β-configured glycosidic bonds, with its putative repeating unit depicted in Figure 3G. Lu et al. systematically analyzed and summarized the structural characteristics and bioactivities of *G. lucidum* polysaccharides through a comprehensive literature review [44]. They reported that the main chains of *G. lucidum* polysaccharides are predominantly composed of β-D-Glcp with minor amounts of α-D-Galp. Notably, the presence of β-D-Glcp in the side chains confers antioxidant activity, while α-L-Fucp in the side chains endows immunomodulatory activity, which is largely consistent with our findings.

In recent years, multi-omics combined approach has become an important strategy to explore the mechanisms of disease development and search for key therapeutic targets, which has been widely used in various studies, especially in the disordered glucose and lipid metabolic syndrome [45,46,47]. The balance of intestinal flora could be significantly disturbed by HFD, and the gut microbiota dysbiosis are considered to be closely associated with the onset and progression of hyperlipidemia, diabetes, obesity, cardiovascular disease, and other related metabolic diseases. In this study, the intervention of HGLP partially restored the unbalanced gut microbial composition and structure triggered by HFD. As the dominant microbes at the phylum level, the roles of Firmicutes and Bacteroidetes are significant in the regulation of host metabolism involving carbohydrates, bile acids, and lipids [48]. Magne et al. found that the intestinal flora of obese patients was rich in Firmicutes, whereas those of thinner patients were rich in Bacteroidetes [49]. The increased Firmicutes/Bacteroidetes ratio is commonly regarded as a marker of obesity, cardiovascular disease, fatty liver, and other disordered lipid metabolism diseases, which could be reversed by exercise or dietary intervention [50,51,52]. Our results showed a marked elevation of Firmicutes/Bacteroidetes ratio induced by HFD, and a decrease in Firmicutes/Bacteroidetes ratio after the treatment of HGLP. In line with our findings, Wang et al. also observed that matcha could observably reduce the relative abundance of Deferribacteres to mitigate obesity and relevant metabolic disorders [53]. *Helicobacter* was tightly associated with the systemic inflammation and high risk of obesity. A considerably higher level of *Helicobacter* was found in the hyperlipidemic mice, implying it was a possibly biomarker of HFD-induced microbiota disorder. However, the decreased abundance of *Helicobacter* in the HGLP group relative to the MC group was consistent with the previous results of Gong et al. [54]. *Bacteroides* was the dominant microbes at the genus level in ApoE^−/−^ mice, suggesting the increase in *Bacteroides* may be related to the occurrence of dyslipidemia, whereas HGLP treatment ulteriorly increased its relative abundance, which may be because of the metabolic ability to dietary polysaccharides of *Bacteroides*, in line with the previous report [55]. Compared with MC group, HGLP increased the relative abundance of *Prevotellaceae_UCG_001*, *Lachnospiraceae_NK4A136_group*, and *Bacteroides* at the genus level. Similarly, Lin et al. also discovered that arabinoxylan could result in the higher relative abundance of *Prevotellaceae_UCG_001*, *Lachnospiraceae_NK4A136_group*, and *Bacteroides* to alleviate obesity by regulating gut microbiota [56]. In the present study, we observed that HFD enriched Actinobacteria, which is involved in lipid metabolism and positively correlated with body mass index (BMI), and the HGLP group enriched *Peptococcaceae_unclassified*, which were in accord with the findings of Chen et al. and Li et al. [57,58].

In this study, we found the 131 differential metabolites were intensively connected with hyperlipidemia, which markedly influenced caffeine metabolism, tryptophan metabolism, staurosporine biosynthesis, nicotinate and nicotinamide metabolism, histidine metabolism, and indole alkaloid biosynthesis pathways. Our current findings indicated a significant reduction in caffeine levels within the caffeine metabolism pathways in the MC group. Moderate caffeine intake could improve fat oxidation via AMPKα-LXRα/SREBP-1c signaling pathway, thus relieving lipid metabolism disorders induced by HFD, and the increased concentration of caffeine was positively correlated with reduced visceral fat accumulation, implying that caffeine may have the potential ability to promote fat burning [59,60,61]. In the MC group, the concentration of tryptophan increased remarkably, which took part in tryptophan metabolism, staurosporine biosynthesis, and indole alkaloid biosynthesis pathways. Tryptophan restrictions promoted a energy expenditure mediated by the sympathetic nervous system, therefore playing the anti-obesity role [62]. The intervention of HGLP obviously altered the levels of 44 differential metabolites, which further mainly influenced amino acid metabolism (phenylalanine metabolism, glycine, serine and threonine metabolism, tryptophan metabolism) and lipid metabolism (primary bile acid biosynthesis). Obesity and metabolic disorders are significantly influenced by the amino acid metabolism of gut microbes, which generate a variety of metabolites such as short-chain and branched-chain fatty acids. In contrast to the MC group, HGLP treatment increased the concentration of 5-hydroxyindoleacetic acid, which further promoted tryptophan metabolism pathways. Hu et al. found that Pu-erh tea could mitigate obesity caused by circadian rhythm disorder-induced obesity, and the potential mechanism maybe related to the increased tryptophan metabolism pathway in serum [46]. Our study showed the remarkable concentration changes in hippuric acid, dihydro-3-coumaric acid, and phenylacetylglycine participating in phenylalanine metabolism, as well as ectoine and dimethylglycine participating in serine and threonine metabolism after HGLP treatment. It was suggested that HGLP may restore the balance of lipid metabolism by regulating amino acid metabolism pathway, which was in line with the findings reported by Jiao et al. [29]. Bile acids, which are crucial signaling molecules and regulators for lipid, glucose, and energy metabolism, have been demonstrated to affect host lipid metabolism via the farnesoid X receptor [26]. Notably, we further found that HGLP significantly regulated primary bile acid biosynthesis pathway, while ginsenoside Rc also modulated primary bile acid biosynthesis pathway of fecal metabolites to ameliorate atherosclerosis and the accompanied cardiovascular diseases [11].

## 5. Conclusions

Overall, our findings indicated that GLP was a homogeneous pyranose ring with the molecular weight of 12.53 kDa linked by α-type and β-type glycosidic bonds, and its possible repeat units of GLP were predicted. GLP effectively regulated the composition and structure of intestinal flora and significantly increased the abundance of energy metabolism and cell growth and death pathways to improve intestinal flora disorder. In addition, GLP had obvious influences on fecal metabolites profile via modulating the amino acid metabolism and lipid metabolism pathways against hyperlipidemia.

## Figures and Tables

**Table 1:** Methylation analysis of GLP.

Linkages Types	Methylated Glucoside	M_W_ (Da)	Relative Molar Ratio (%)
t-Fuc(p)	1,5-di-O-acetyl-6-deoxy-2,3,4-tri-O-methyl fucitol	293	6.157
t-Man(p)	1,5-di-O-acetyl-2,3,4,6-tetra-O-methyl mannitol	323	5.472
t-Gal(p)	1,5-di-O-acetyl-2,3,4,6-tetra-O-methyl galactitol	323	2.236
3-Glc(p)	1,3,5-tri-O-acetyl-2,4,6-tri-O-methyl glucitol	351	2.361
2-Gal(p)	1,2,5-tri-O-acetyl-3,4,6-tri-O-methyl galactitol	351	3.836
6-Glc(p)	1,5,6-tri-O-acetyl-2,3,4-tri-O-methyl glucitol	351	8.231
4-Glc(p)	1,4,5-tri-O-acetyl-2,3,6-tri-O-methyl glucitol	351	5.681
6-Gal(p)	1,5,6-tri-O-acetyl-2,3,4-tri-O-methyl galactitol	351	50.675
3,6-Glc(p)	1,3,5,6-tetra-O-acetyl-2,4-di-O-methyl glucitol	379	1.919
4,6-Glc(p)	1,4,5,6-tetra-O-acetyl-2,3-di-O-methyl glucitol	379	0.612
2,6-Gal(p)	1,2,5,6-tetra-O-acetyl-3,4-di-O-methyl galactitol	379	12.819

**Table 2:** ^1^H and ^13^C chemical shifts in glycosyl residues in GLP.

Code	Glycosyl Residues	Chemical Shifts (ppm)					
		H1/C1	H2/C2	H3/C3	H4/C4	H5/C5	H6a,6b/C6
A	→6)-α-D-Gal*p*-(1→	4.93	3.78	4.01	3.77	3.81	3.84, 3.61
		97.87	71.65	69.49	68.27	69.41	66.54
B	→2,6)-α-D-Gal*p*-(1→	5	3.75	3.99	3.78	3.95	4.14, 3.65
		97.9	77.98	68.31	71.75	69.53	66.79
C	→6)-β-D-Glc*p*-(1→	4.46	3.26	3.51	3.4	3.63	4.12, 3.92
		102.91	73.05	71.05	69.46	72.53	67.22
D	α-L-Fuc*p*-(1→	5.01	3.72	3.79	4.08	4.15	1.16
		101.49	68.4	69.45	69.18	68.82	15.66
E	→4)-β-D-Glc*p*-(1→	4.67	3.31	3.95	3.42	n.d	3.84, 3.68
		102.77	73.33	70.93	75.59	n.d	60.74
F	α-D-Man*p*-(1→	5.08	3.77	n.d	n.d	n.d	3.57
		96.06	68.68	n.d	n.d	n.d	66.8

## Data Availability

The original contributions presented in this study are included in the article/Supplementary Materials. Further inquiries can be directed to the corresponding authors.

## Supplementary Materials

The following supporting information can be downloaded at https://www.mdpi.com/article/10.3390/biom16010153/s1, Figure S1: Alpha diversity analysis of gut microbiota in mice; Figure S2: Predictive functional abundance heatmap of KEGG pathway of microbial communities by PICRUSt2; Figure S3: Chemical taxonomy plots of differential metabolites between (A) MC vs. NC groups, and (B) HGLP vs. MC groups; Figure S4: Clustering heatmap analysis of differential metabolites in feces between (A) MC vs. NC groups and (B) HGLP vs. MC groups; Table S1: The differential metabolites and trends between MC and NC groups; Table S2: The differential metabolites and trends between HGLP and MC groups.

## Abbreviations

FT-IR: Fourier transform infrared spectrometry; GC-MS: Gas chromatography-mass spectrometry; GLP: *Ganoderma lucidum* polysaccharides; GPC: Gel permeation chromatography; LC-MS: Liquid chromatography-mass spectrometry; LDA: Linear discriminant analysis; LEfSe: LDA effect size; Mn: Number-average molecular weight; Mw: Weight-average molecular weight; NMR: Nuclear magnetic resonance; OPLS-DA: Orthogonal partial least squares discriminant analysis; OTU: Operational taxonomic units; PCA: Principal component analysis; PCoA: Principal coordinate analysis; PLS-DA: Partial least squares discriminant analysis; QC: Quality control; SEM: Scanning electron microscope; VIP: Variable importance for the projection.
