# Supplementary material for: Study on the Mechanism of Ganoderma lucidum Polysaccharides for Ameliorating Dyslipidemia via Regulating Gut Microbiota and Fecal Metabolites
Source: Biomolecules. 2026 Jan 14;16(1):153. doi: 10.3390/biom16010153 (PMC12839207; doi:10.3390/biom16010153)
Supplement: Supplementary file 1 [file biomolecules-16-00153-s001.zip › biomolecules-4062408-supplementary.pdf]

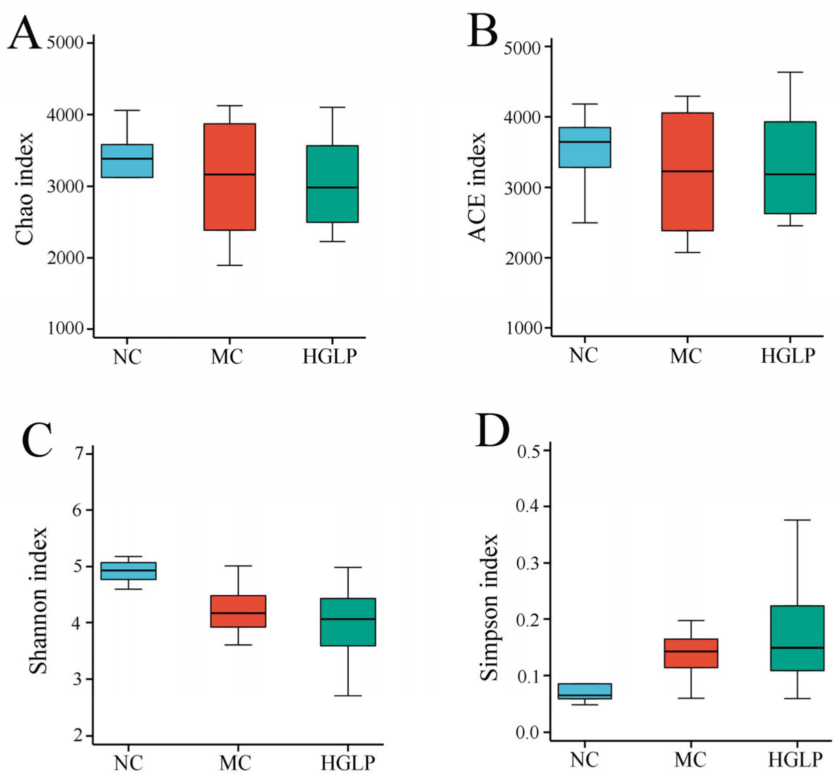

Figure S1 Alpha diversity analysis of gut microbiota in mice. (A) Chao index, (B) ACE index, (C) Shannon index and (D) Simpson index. Wilcoxon rank-sum test was used to analysis the differences between groups in Alpha diversity indices, and $P < 0.05$ was assessed as statistically significant difference.

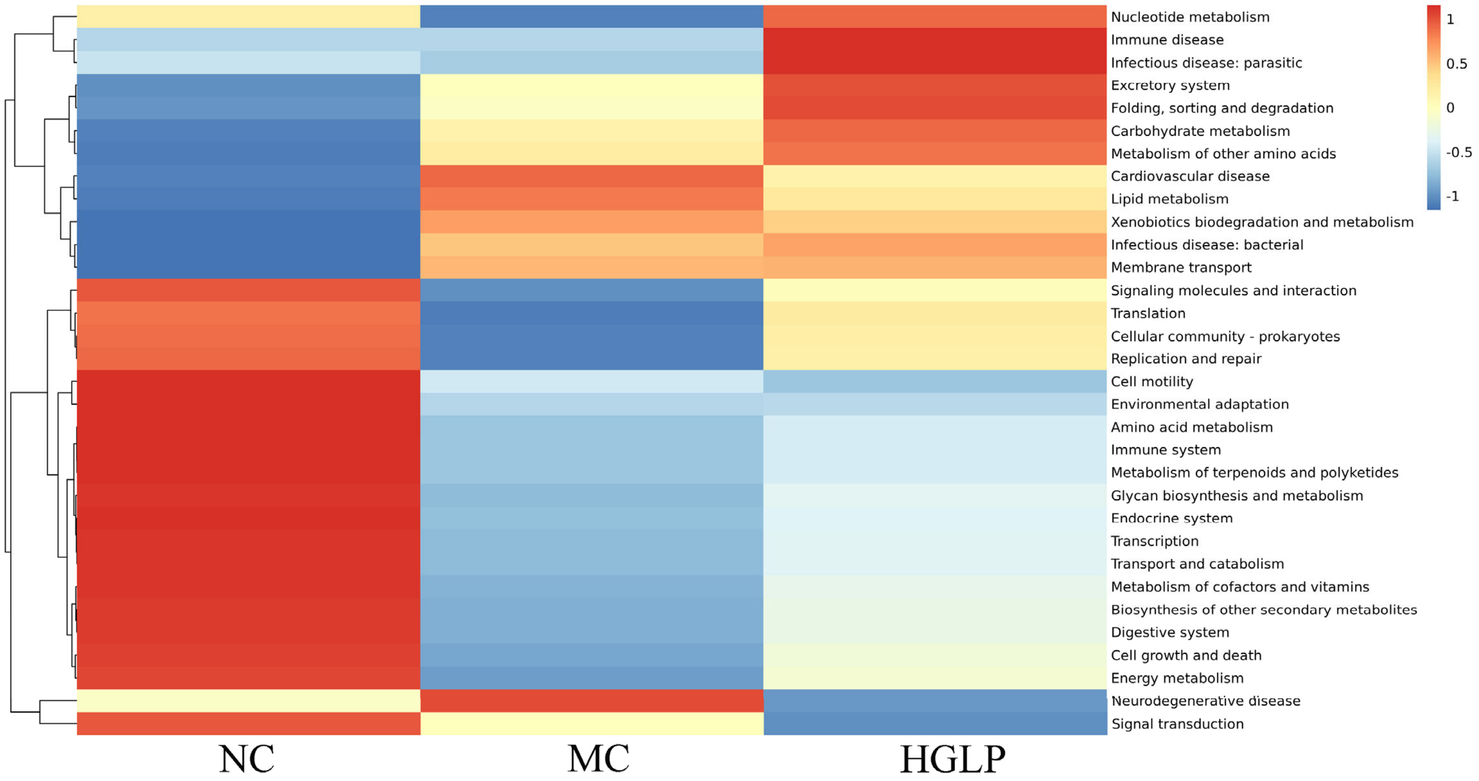

Figure S2 Predictive functional abundance heatmap of KEGG pathway of microbial communities by PICRUSt2.

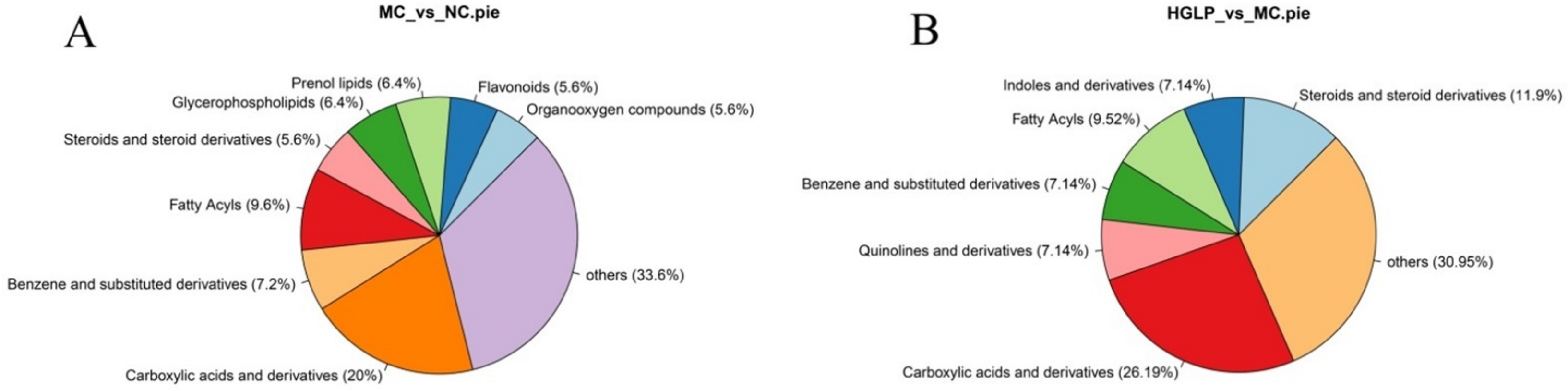

Figure S3 Chemical taxonomy plots of differential metabolites between (A) MC vs. NC groups, and (B) HGLP vs. MC groups.

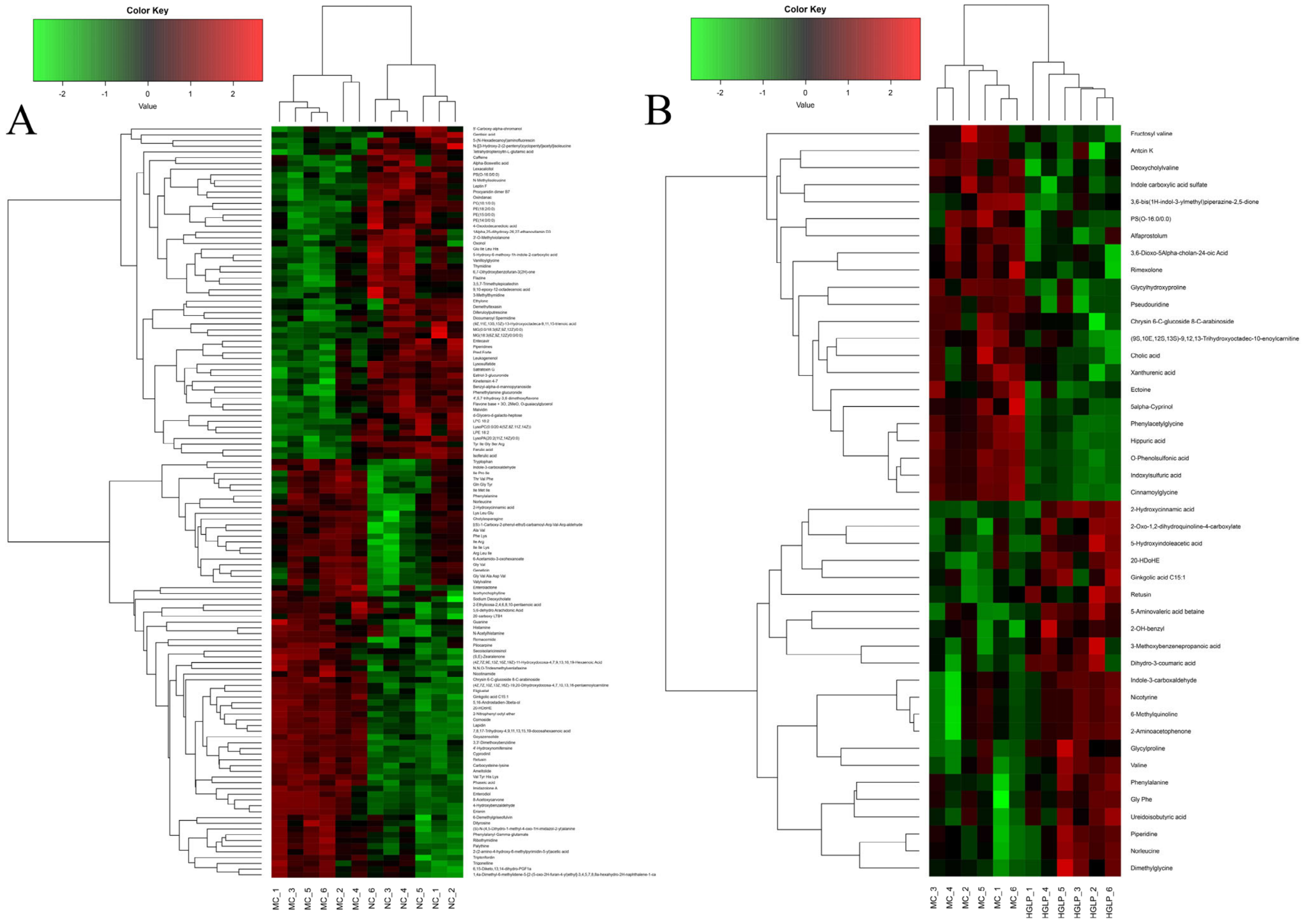

Figure S4 Clustering heatmap analysis of differential metabolites in feces between (A) MC vs. NC groups and (B) HGLP vs. MC groups. The color of the square depicted the relative expression size of differential metabolites in each sample. Red color indicated that the expression of metabolite was increased, while green color indicated that the expression of metabolite was decreased.

Table S1 The differential metabolites and trends between MC and NC groups.

Mode	Metabolite	Retention time (min)	VIP	FC	p value	MC vs. NC
pos	1,4a-Dimethyl-6-methylidene-5-[2-(5-oxo-2H-furan-4-yl)ethyl]-3,4,5,7,8,8a-hexahydro-2H-naphthalene-1-carboxylic acid	6.621117	1.127153	1.972609	0.006885	↑
pos	Tripterifordin	6.674667	1.154832	2.235427	0.000798	↑
neg	Valylvaline	1.0811	1.372668	2.134466	0.042664	↑
pos	Erianin	5.678383	6.428463	69.03765	0.002157	↑
neg	Enterodiol	6.118933	13.09201	21.30996	0.002984	↑
pos	N,N,O-Tridesmethylvenlafaxine	8.766917	1.091313	5.837399	0.024582	↑
pos	Arg Leu Ile	1.342383	2.042229	4.145881	0.013612	↑
pos	Ameltolide	7.229883	1.375105	9.74329	9.13E-06	↑
pos	Ile Met Ile	5.066533	1.302718	2.117916	0.021817	↑
pos	Ile Ile Lys	1.40385	1.726365	3.025884	0.00914	↑
pos	Thr Val Phe	4.481633	1.097662	1.779732	0.038971	↑
pos	Imidazolone A	0.865633	2.317742	7.106027	0.000575	↑
pos	Cyprodinil	4.827683	1.762934	33.90281	0.000381	↑
pos	Pilocarpine	1.014017	1.0351	2.625976	0.006779	↑
pos	2-(2-amino-4-hydroxy-6-methylpyrimidin-5-yl)acetic acid	0.837117	1.78101	2.183723	0.004126	↑
pos	(4Z,7Z,9E,13Z,16Z,19Z)-11-Hydroxydocosa-4,7,9,13,16,19-Hexaenoic Acid	9.527433	1.321673	2.238057	0.001633	↑
pos	Ala Val	0.987133	2.078843	1.979194	0.024772	↑
pos	7,8,17-Trihydroxy-4,9,11,13,15,19-docosahexaenoic acid	7.294267	1.775531	5.943085	7.84E-06	↑

pos	6,15-Diketo,13,14-dihydro-PGF1a	6.11115	1.447669	2.138846	0.007232	↑
neg	[(S)-1-Carboxy-2-phenyl-ethyl]-carbamoyl-Arg-Val-Arg-aldehyde	4.981733	1.228158	2.86018	0.021813	↑
neg	Carbocysteine-lysine	0.787783	1.817686	8.618086	9.22E-07	↑
pos	Palythine	0.78395	2.302226	3.654957	0.000323	↑
neg	20-carboxy-LTB4	7.001767	1.43246	2.372438	0.013247	↑
neg	Retusin	6.082317	1.007588	4.240102	5.14E-05	↑
neg	Ginkgolic acid C15:1	9.7888	1.400597	3.373156	1.59E-05	↑
pos	N-Acetylhistamine	0.765983	4.800997	4.024183	0.046056	↑
pos	Ile Pro Ile	4.7134	1.330127	2.451401	0.017384	↑
pos	(S,E)-Zearalenone	5.385983	1.238536	5.338896	0.01584	↑
pos	Trigonelline	0.819083	1.706968	1.865624	0.040982	↑
pos	Ribothymidine	0.78395	2.021055	2.853942	0.00385	↑
neg	20-HDoHE	9.4248	1.676506	3.90322	2.14E-05	↑
neg	Gly Val Ala Asp Val	3.935217	1.030102	2.681435	0.020545	↑
pos	8-Acetoxycarvone	5.678383	1.422397	22.86303	0.001679	↑
neg	Secoisolariciresinol	6.131983	1.272378	2.64831	0.008294	↑
pos	Remacemide	8.740333	3.294722	82.94282	0.000569	↑
neg	Phaseic acid	8.2775	1.484713	4.849864	7.42E-05	↑
pos	Goyazensolide	5.58595	1.021338	15.36376	0.000411	↑
pos	3,3'-Dimethoxybenzidine	6.465	1.083208	100.77	0.000414	↑
pos	2-Hydroxycinnamic acid	1.023033	2.939575	1.30819	0.019443	↑
pos	(S)-N-(4,5-Dihydro-1-methyl-4-oxo-1H-imidazol-2-yl)alanine	0.903917	1.305383	2.625875	0.016129	↑
pos	4'-Hydroxynomifensine	7.981183	1.211067	62.60617	0.000223	↑

pos	Phenylalanyl-Gamma-glutamate	1.342383	1.0372	3.770994	0.001051	↑
neg	Cholylasparagine	4.647633	1.019332	2.547929	0.012683	↑
pos	Lys Leu Glu	0.950933	1.209252	2.233507	0.02769	↑
pos	Phe Lys	0.894517	2.185125	2.877464	0.00272	↑
pos	4-Hydroxybenzaldehyde	5.678383	1.766028	21.94123	0.001277	↑
pos	Dityrosine	0.95995	1.117838	2.938609	0.021002	↑
pos	Histamine	0.691017	3.031164	7.189832	0.031663	↑
pos	Cornoside	4.543117	1.142714	8.69735	0.000127	↑
pos	Indole-3-carboxaldehyde	3.643967	1.270312	1.800452	0.021767	↑
pos	2-Nitrophenyl octyl ether	6.853767	1.144833	5.255023	8.07E-05	↑
pos	6-Demethylgriseofulvin	0.6262	1.310676	1.608451	0.030181	↑
pos	Lapidin	6.1235	1.927382	11.28017	1.94E-05	↑
pos	Ile Arg	0.765983	2.519189	2.814575	0.002649	↑
neg	Val Tyr His Lys	6.848517	1.018801	1499.425	0.036663	↑
pos	Eliglustat	7.515567	1.20937	24.27071	0.000583	↑
pos	Nicotinamide	1.353767	1.096791	6.729539	0.01381	↑
pos	6-Acetamido-3-oxohexanoate	0.95995	1.987208	1.628348	0.006786	↑
pos	Geneticin	4.407467	1.01503	2.269015	0.043061	↑
pos	Guanine	0.950933	2.859873	1.640344	0.028037	↑
pos	Norleucine	1.031733	5.065424	1.451041	0.03991	↑
pos	(4Z,7Z,10Z,13Z,16Z)-19,20-Dihydroxydocosa-4,7,10,13,16-pentaenoylcarnitine	6.356817	1.073189	16.58063	6.89E-05	↑
neg	5,16-Androstadien-3beta-ol	9.164017	1.562699	4.049101	5.91E-05	↑
pos	Gly Val	1.005183	1.154254	1.815794	0.046431	↑

neg	Phenylalanine	1.65395	3.443322	1.725821	0.029818	↑
neg	Isorhynchophylline	5.70085	1.808464	4.444625	0.006496	↑
pos	Gln Gly Tyr	1.092033	1.044878	1.97683	0.012138	↑
neg	Tryptophan	3.646683	5.530637	2.006237	0.011095	↑
neg	5,6-dehydro Arachidonic Acid	10.69223	2.643523	1.700562	0.044806	↑
neg	Enterolactone	6.907267	6.017864	2.257311	0.027014	↑
neg	2-Ethylicosa-2,4,6,8,10-pentaenoic acid	11.31257	1.020806	2.039784	0.017279	↑
neg	Chrysin 6-C-glucoside 8-C-arabinoside	6.497883	1.068977	9.706239	0.002719	↑
neg	Sodium Deoxycholate	14.8757	1.263192	3.645658	0.00083	↑
neg	Isoferulic acid	5.41885	5.20441	0.263068	0.000468	↓
pos	MG(18:3(6Z,9Z,12Z)/0:0/0:0)	8.781067	1.269936	0.495065	0.03598	↓
neg	3-Methylthymidine	1.000767	1.121049	0.391785	0.035548	↓
pos	3,5,7-Trimethylepicatechin	3.842233	1.597147	0.171568	0.036213	↓
pos	Flazine	6.451567	1.568317	0.208602	0.022729	↓
pos	4-Oxododecanedioic acid	4.966167	1.31418	0.381292	0.031863	↓
neg	Oxonol	4.887767	2.034588	0.367682	0.037726	↓
pos	(9Z,11E,13S,15Z)-13-Hydroxyoctadeca-9,11,15-trienoic acid	8.971383	1.091412	0.55811	0.005364	↓
pos	9,10-epoxy-12-octadecenoic acid	8.546033	1.172887	0.432828	0.041045	↓
pos	N-[[3-Hydroxy-2-(2-pentenyl)cyclopentyl]acetyl]isoleucine	7.089533	1.207897	0.441584	0.048482	↓
pos	6,7-Dihydroxybenzofuran-3(2H)-one	0.846917	1.405787	0.270055	0.003546	↓
neg	Oxindanac	5.568117	1.258336	0.103493	0.003317	↓
neg	Leptin F	4.746283	2.480555	0.269337	0.004416	↓
pos	MG(0:0/18:3(6Z,9Z,12Z)/0:0)	9.420317	2.277171	0.439566	0.028059	↓

pos	Malvidin	6.803483	1.851168	0.37159	0.000106	↓
neg	Vanilloylglycine	3.466083	1.822712	0.418716	0.043302	↓
pos	3'-O-Methylviolanone	5.3993	1.705917	0.267603	0.008419	↓
pos	Glu Ile Leu His	4.007117	1.505502	0.459383	0.013384	↓
pos	Phenethylamine glucuronide	0.97755	2.069309	0.323912	0.014153	↓
neg	4',5,7-trihydroxy-3,6-dimethoxyflavone	6.812233	1.823185	0.387144	0.001657	↓
pos	PE(14:0/0:0)	8.289717	1.882801	0.244556	0.031368	↓
neg	5-(N-Hexadecanoyl)aminofluorescin	5.627817	1.148898	0.163666	0.004188	↓
neg	Gentisic acid	3.5964	1.779099	0.343069	0.046421	↓
pos	LPE 18:2	8.716183	1.630875	0.214174	0.049569	↓
neg	PE(15:0/0:0)	8.741133	2.783333	0.208363	0.027038	↓
pos	Ferulic acid	5.425183	2.012792	0.293997	0.000869	↓
pos	Flavone base + 3O, 2MeO, O-guaiacylglycerol	6.725783	1.08192	0.274359	0.001614	↓
pos	Leukogenenol	3.917517	1.977202	0.209845	0.001545	↓
pos	Dicoumaroyl Spermidine	4.789233	3.999021	0.328743	0.02427	↓
pos	Demethyltexasin	5.7557	1.897705	0.338758	0.009794	↓
neg	5-Hydroxy-6-methoxy-1h-indole-2-carboxylic acid	3.1502	1.738819	0.322323	0.047896	↓
pos	N-Methylisoleucine	1.005183	1.610758	0.093308	0.013335	↓
neg	5'-Carboxy-alpha-chromanol	8.463483	1.064253	0.419768	0.016372	↓
pos	1Alpha,25-dihydroxy-26,27-ethanovitamin D3	9.6075	1.139252	0.564572	0.032657	↓
pos	LysoPC(0:0/20:4(5Z,8Z,11Z,14Z))	8.793567	2.145093	0.168068	0.040201	↓
neg	LysoPA(20:2(11Z,14Z)/0:0)	11.95247	1.058956	0.558839	0.030906	↓

neg	Pred Forte	3.9199	1.338046	0.392437	0.006029	↓
pos	Thymidine	0.903917	1.075863	0.391791	0.003985	↓
neg	Estriol-3-glucuronide	3.777417	1.884787	0.216411	0.011282	↓
pos	Diferuloylputrescine	6.030167	5.687253	0.316403	0.005505	↓
pos	Piperidines	1.110517	1.823156	0.177166	0.022646	↓
pos	Benzyl-alpha-d-mannopyranoside	1.378783	1.085911	0.180804	0.002016	↓
pos	Kinetensin 4-7	4.555733	2.236379	0.267601	0.001318	↓
pos	Ethylone	5.859333	1.655238	0.301155	0.016087	↓
pos	PE(18:2/0:0)	8.858667	2.403021	0.241278	0.003547	↓
neg	Procyanidin dimer B7	5.039233	1.401101	0.161143	0.001731	↓
pos	Entecavir	0.819083	1.956039	0.166668	0.029105	↓
pos	Alpha-Boswellic acid	8.781067	1.726312	0.207015	0.016902	↓
pos	PS(O-16:0/0:0)	5.385983	1.765549	0.503974	0.00156	↓
pos	Lysosulfatide	0.885683	1.181037	0.0961	0.000755	↓
pos	d-Glycero-d-galacto-heptose	0.77525	1.053245	0.289053	0.014638	↓
pos	LPC 18:2	8.766917	3.384952	0.171395	0.006643	↓
neg	Tetrahydropteroyltri-L-glutamic acid	4.064767	1.030732	0.177428	0.049033	↓
pos	Lexacalcitol	11.57833	1.51187	0.172474	0.002881	↓
pos	Satratoxin G	0.810317	1.278431	0.151508	0.004718	↓
pos	PC(18:1/0:0)	9.527433	2.035331	0.277681	0.005566	↓
pos	Caffeine	3.389817	2.271621	0.75027	0.0311	↓
neg	Tyr Ile Gly Ser Arg	7.120033	1.044443	0.103058	0.003712	↓

↑indicated that the content of metabolite was up-regulated in MC group when compared with NC group.
↓indicated that the content of metabolite was down-regulated in MC group when compared with NC group.

Table S2 The differential metabolites and trends between HGLP and MC groups.

Mode	Metabolite	Retention time (min)	VIP	FC	p value	HGLP vs. MC
pos	Dimethylglycine	0.77525	1.054979	1.472428	0.049356	↑
neg	Dihydro-3-coumaric acid	5.372383	11.3237	1.616312	0.015164	↑
pos	Valine	0.85665	6.634951	1.696423	0.018697	↑
neg	Retusin	6.082317	1.155787	1.978152	0.037225	↑
neg	Ginkgolic acid C15:1	9.7888	1.216685	1.408806	0.035301	↑
neg	2-OH-benzyl	5.902783	1.364308	1.510673	0.037445	↑
pos	2-Aminoacetophenone	3.643967	1.118061	1.42079	0.02624	↑
neg	20-HDoHE	9.4248	1.5347	1.413244	0.014087	↑
pos	5-Hydroxyindoleacetic acid	5.000767	2.883242	1.260758	0.041146	↑
pos	2-Hydroxycinnamic acid	1.023033	4.413875	1.23696	0.005771	↑
pos	5-Aminovaleric acid betaine	0.792883	3.93368	1.291217	0.008944	↑
neg	3-Methoxybenzenepropanoic acid	5.372383	2.043993	1.54634	0.042578	↑
neg	Ureidoisobutyric acid	0.750967	1.517985	1.612723	0.025271	↑
pos	Indole-3-carboxaldehyde	3.643967	1.811998	1.426129	0.021923	↑
pos	Nicotyrine	3.643967	1.244231	1.384208	0.025838	↑
pos	2-Oxo-1,2-dihydroquinoline-4-carboxylate	2.982667	1.852771	1.334039	0.028406	↑
pos	Gly Phe	2.713467	1.78697	1.385769	0.029991	↑
pos	Norleucine	1.031733	7.120826	1.288503	0.028912	↑
pos	Piperidine	1.031733	2.416333	1.300958	0.042154	↑
pos	6-Methylquinoline	3.643967	1.31398	1.428422	0.027022	↑
pos	Glycylproline	0.810317	1.526852	1.54213	0.022131	↑

neg	Phenylalanine	1.65395	6.090616	1.6089	0.012585	↑
neg	Cinnamoylglycine	5.677717	2.169624	0.061716	0.002598	↓
pos	Alfaprostolum	9.367233	1.215458	0.507276	0.010961	↓
pos	Xanthurenic acid	3.766833	1.598086	0.335201	0.017277	↓
neg	Indoxylsulfuric acid	5.06565	5.883054	0.040771	0.004743	↓
neg	Pseudouridine	0.904883	1.425843	0.477448	0.01631	↓
neg	Glycylhydroxyproline	0.925283	1.848398	0.35971	0.000747	↓
pos	3,6-bis(1H-indol-3-ylmethyl)piperazine-2,5-dione	6.148817	1.370014	0.170799	0.049175	↓
pos	Cholic acid	7.6989	9.501801	0.441271	0.044033	↓
neg	Hippuric acid	4.60205	4.020291	0.068784	0.002347	↓
neg	O-Phenolsulfonic acid	4.734483	5.375274	0.047199	0.001711	↓
neg	Indole carboxylic acid sulfate	4.441417	5.709134	0.234672	0.019651	↓
pos	(9S,10E,12S,13S)-9,12,13-Trihydroxyoctadec-10-enoylcarnitine	7.19205	1.1619	0.557529	0.013416	↓
pos	Ectoine	0.78395	1.563763	0.369807	0.022392	↓
pos	Rimexolone	7.721883	1.677631	0.674419	0.013233	↓
pos	5alpha-Cyprinol	8.135267	2.607934	0.280848	0.035614	↓
pos	Phenylacetylglycine	4.942283	1.841162	0.052476	0.019573	↓
pos	Deoxycholylvaline	7.92975	1.189012	0.52623	0.001283	↓
pos	Fructosyl valine	0.885683	1.323697	0.392648	0.044361	↓
neg	Antcin K	9.347217	1.589322	0.546233	0.023018	↓
pos	3,6-Dioxo-5Alpha-cholan-24-oic Acid	7.659317	2.608895	0.672959	0.005389	↓
pos	PS(O-16:0/0:0)	5.385983	1.235208	0.664326	0.016686	↓

neg	Chrysin 6-C-glucoside 8-C-arabinoside	6.497883	1.081271	0.337736	0.018768	↓

↑indicated that the content of metabolite was up-regulated in HGLP group when compared with MC group.
↓indicated that the content of metabolite was down-regulated in HGLP group when compared with MC group.
